# A Fluid–Structure Interaction Study of Different Bicuspid Aortic Valve Phenotypes Throughout the Cardiac Cycle

**DOI:** 10.3389/fphys.2021.716015

**Published:** 2021-07-26

**Authors:** Wentao Yan, Jianming Li, Wenshuo Wang, Lai Wei, Shengzhang Wang

**Affiliations:** ^1^Department of Aeronautics and Astronautics, Fudan University, Shanghai, China; ^2^Department of Vascular Surgery, Zhongshan Hospital Affiliated to Fudan University, Shanghai, China; ^3^Institute of Biomedical Engineering Technology, Academy for Engineering and Technology, Fudan University, Shanghai, China

**Keywords:** bicuspid aortic valve, different phenotypes, fluid–structure Interaction, hemodynamics, valve mechanics

## Abstract

The bicuspid aortic valve (BAV) is a congenital malformation of the aortic valve with a variety of structural features. The current research on BAV mainly focuses on the systolic phase, while ignoring the diastolic hemodynamic characteristics and valve mechanics. The purpose of this study is to compare the differences in hemodynamics and mechanical properties of BAV with different phenotypes throughout the cardiac cycle by means of numerical simulation. Based on physiological anatomy, we established an idealized tricuspid aortic valve (TAV) model and six phenotypes of BAV models (including Type 0 a–p, Type 0 lat, Type 1 L–R, Type 1 N-L, Type 1 R-N, and Type 2), and simulated the dynamic changes of the aortic valve during the cardiac cycle using the fluid–structure interaction method. The morphology of the leaflets, hemodynamic parameters, flow patterns, and strain were analyzed. Compared with TAV, the cardiac output and effective orifice area of different BAV phenotypes decreased certain degree, along with the peak velocity and mean pressure difference increased both. Among all BAV models, Type 2 exhibited the worst hemodynamic performance. During the systole, obvious asymmetric flow field was observed in BAV aorta, which was related to the orientation of BAV. Higher strain was generated in diastole for BAV models. The findings of this study suggests specific differences in the hemodynamic characteristics and valve mechanics of different BAV phenotypes, including different severity of stenosis, flow patterns, and leaflet strain, which may be critical for prediction of other subsequent aortic diseases and differential treatment strategy for certain BAV phenotype.

## Introduction

**FIGURE 1:**
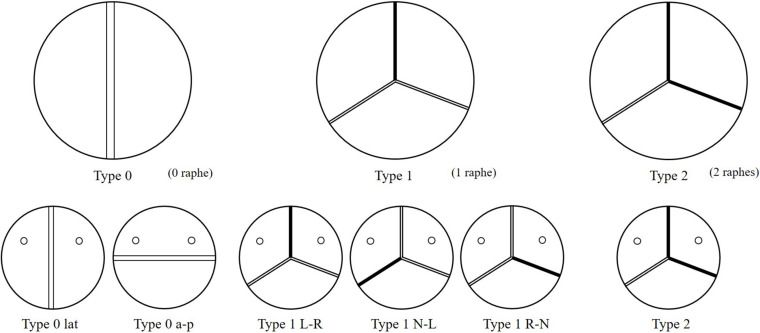
Schematic of six phenotypes of BAV models.

Bicuspid aortic valve (BAV) malformation is a relatively common congenital heart valve disease, which is mainly manifested by abnormal changes in the number of valves. The incidence of BAV in general population is about 0.5–2%, and the incidence of men is greater than that of women (McKellar et al., 2010; Siu and Silversides, 2010). A high genetic tendency of BAV was also found (Aggarwal et al., 2012; Laforest and Nemer, 2012). Studies have shown that the development of the BAV causes the two leaflets of the aortic valve to fail to separate from each other and fuse together. According to the Sievers and Schmidtke (2007) classification method, BAV can be categorized as Type 0, Type 1, and Type 2 based on the spatial position and number of raphes. Furthermore, Type 0 can be divided into two subtypes, anterior–posterior (Type 0 a–p) and lateral (Type 0 lat), according to the orientation. Type 1 can also be classified into three subtypes, left–right fusion (Type 1 L–R), non-coronary-left fusion (Type 1 N-L), non-coronary-right fusion (Type 1 R-N), depending on the leaflet fusion. Type 2 usually happens with left–right fusion and non-coronary-right fusion simultaneously. Phenotypes of BAV are shown in Figure 1.

Compared with tricuspid aortic valve (TAV), patients with BAV have a higher incidence of aortic diseases, which can be mainly divided into valve disease and aortic vascular disease, including aortic valve stenosis, aortic regurgitation, aortic dissection, ascending aortic dilation, and infective endocarditis (Kari et al., 2012). Besides the genetic factors, the biomechanical and hemodynamic changes caused by the BAV malformation also play an important role in aortic diseases (Conti et al., 2010; Sievers and Sievers, 2011; Sun et al., 2012). However, how different phenotypes of BAVs affect the hemodynamics and cause related aortic diseases, it is still not yet understood fully and requires a systematic research. These issues have always been concerned by researchers, who have studied it through clinical analysis, *in vitro* experiment and computational simulation (Saikrishnan et al., 2012; Bahraseman et al., 2014). The computational simulation method, especially the fluid–structure interaction (FSI) analysis, can simulate the function of the leaflets well and predict the hemodynamics of the aorta, and was used by more and more researchers in heart valve problems (Bahraseman et al., 2013; Chen and Luo, 2018; Emendi et al., 2020).

Many studies had been carried out in recent years, based on general models or patient-specific models, different leaflet phenotypes, dilated or non-dilated aorta, and different FSI methods. Gilmanov and Sotiropoulos (2016) compared the aortic hemodynamic performance of TAV and BAV (Type 1 R–L) with the FSI analysis using commercial finite element analysis software LS-DYNA. The results showed great differences in flow patterns between TAV and BAV in aorta, mainly in the dynamics of the formation and rupture of the aortic valve vortex ring, the flow field of the ascending aorta, and the wall shear stress (WSS) of the valve. Cao and Sucosky (2017) compared the differences of the leaflet stress and deformation of TAV and BAV (Type 0 a–p, Type 1 RL) by the index of TSM, oscillatory shear index (OSI), TSG, and leaflet stretch with the FSI analysis commercial software ANSYS. The results indicated the abnormal hemodynamic stress caused by BAV, and the degree and regional characteristics of the abnormality are morphotype-dependent. Cao et al. (2017) also did a quantitative analysis on the hemodynamic performance in non-dilated TAV and BAV (Type 1 R–L, Type 1 R-N, and Type 1 N-L) aortas. The results showed strong abnormal hemodynamics in the Type 1 non-dilated aorta, and the coexistence of abnormal direction and position of the wall stress lead to the dilation of the Type 1 non-dilated aorta. Oliveira et al. (2020) studied the hemodynamics and wall stress of the patient-specific BAV (Type 0, Type 1 R–L, and Type 1 R-N) in the dilated aorta during systole using commercial software COMSOL. Compared with the non-dilated aorta, abnormal hemodynamics would also appear in the dilated aorta, which could be demonstrated by the characteristics of blood flow and reginal WSS. de Oliveira et al. (2020) studied the hemodynamics and leaflet mechanical properties of TAV and all phenotypes of BAV (Type 0, Type 1, and Type 2) only in the systole period. The results showed that Type 0 produced the best hemodynamics and mechanical properties, and the raphe of Type 1 changed the position of abnormal hemodynamic characteristics, while Type 2 inhibited the development of blood flow, and the leaflet stress and hemodynamic parameters were the worst.

The above studies had mentioned the abnormal performance of BAVs in flow patterns and stress during the systole period. However, the aortic valve suffers a higher pressure in the reverse blood flow in diastole than in systole. No systematical studies about all phenotypes of BAVs throughout the cardiac cycle were performed yet, especially in the diastolic period. The hemodynamic effect of the reverse blood flow on the aortic sinus and the valve is not yet known (Gilmanov and Sotiropoulos, 2016). Based on this, our study aims to analyze the hemodynamics and mechanical characteristics of different phenotypes of BAVs (including Type 0 a–p, Type 0 lat, Type 1 L–R, Type 1 N-L, Type 1 R-N, and Type 2) throughout the cardiac cycle, and try to find out the potential links between various phenotypes and the occurrence of aortic diseases.

## Methodology

### Modeling Approaches

**FIGURE 2:**
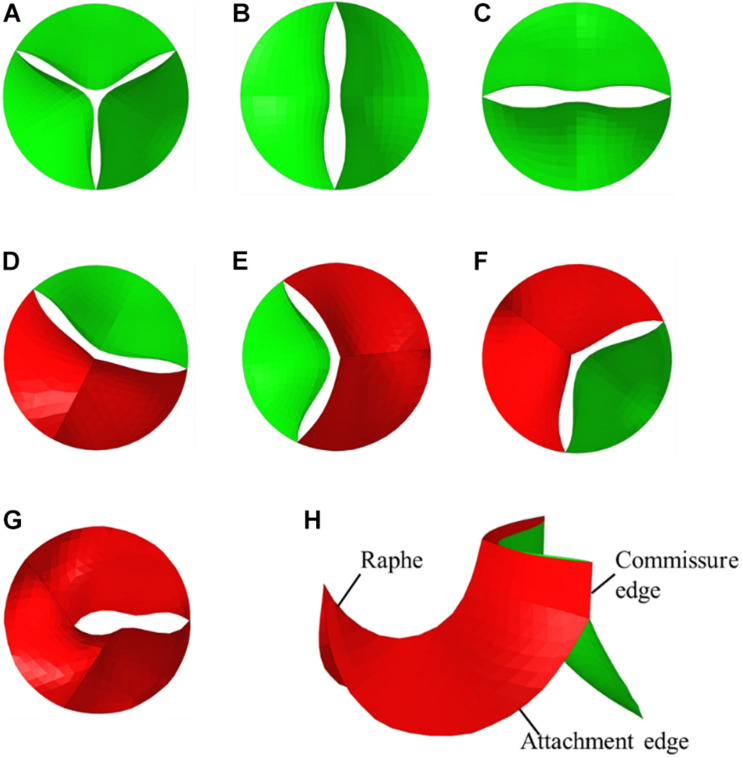
Geometries of TAV and BAV **(A)** TAV, **(B)** Type 0 a–p, **(C)** Type 0 lat, **(D)** Type 1 L–R, **(E)** Type 1 N-L, **(F)** Type 1 R-N, **(G)** Type 2, **(H)** isometric view of BAV model.

**FIGURE 3:**
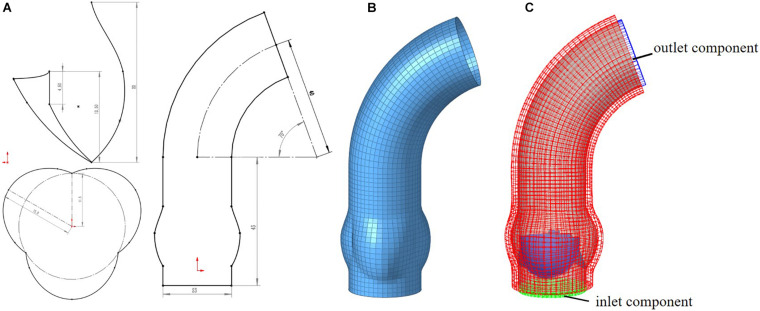
Computational model for aorta **(A)** 2D-sketch of aorta, **(B)** mesh model of aorta, **(C)** entire FSI computational model.

Idealized models based on physiology were set up including a TAV model and six BAV models representing: Type 0 with anterior–posterior (a–p) and lateral (lat) orientations, Type 1 with L–R, N-L, and R-N leaflet fusion, respectively, and Type 2 with L–R/R-N leaflet fusion, as shown in Figure 2. An “U” shape profile shown in Figure 2H was considered for the fusion raphes in type 1 and type 2 to represent congenital malformation, based on the physiological anatomy and previous studies (Sievers and Schmidtke, 2007; Cao and Sucosky, 2017; Pasta et al., 2017). A “half-open” position was chosen as the initial geometries as the “stress-free” state for all the aortic valve models. The aorta was constructed from the left ventricle outflow tract to the aortic arch, and all the branches were neglected to simplify the computational models. Two sinuses were built in the aortic root for Type 0 models and three sinuses for others. A constant 23 mm diameter was assumed for the virtual valvular ring and the ascending aorta. Under this size, the sinus height and leaflet height were set to 22 and 12.5 mm, respectively, based on a healthy physiological model (Labrosse et al., 2006). The commissure height was set to 4 mm, about 0.4–0.5 times the annular radius (Labrosse et al., 2006; Haj-Ali et al., 2012). Dimensions for the aortic valve and aorta are presented in Figure 3A. Leaflets were constructed with Non-Uniform Rational B-Splines (NURBS) surface, with the same parameters in leaflet height and commissure height for all models. All idealized models were generated using Solidworks 2019 (Dassault Systemes, United States).

Meshing of all geometric models was generated in Hypermesh 19.0 (Altair, United States). One of the computational models is shown in Figure 3. A mesh convergence analysis was performed to determine the fluid mesh size and ensure the computational accuracy. Three mesh sizes were tested in TAV model. The relative errors in cardiac output (CO) and peak velocity between the 1.2 and 1.0 mm size were below 8%, as shown in Table 1, so the fluid domain was meshed with 1.2 mm size high-quality hexahedral elements with a total number of approximate 30,000. A spatial resolution of 1.4 mm was set for the aortic valve, and the aortic wall grid size was set to 1.5 mm. The aortic valve and aortic wall were meshed with triangular and quadrilateral elements, respectively. In order to improve the calculation efficiency, shell element formulation with uniform thickness was adopted for the aortic valve and the aortic wall, and the thickness was 1 mm for the aortic valve and 2 mm for the aortic wall.

**TABLE 1:** Mesh convergence analysis for fluid domain of cardiac output and peak velocity for TAV model.

Mesh size (mm)	CO (L/min)	Peak velocity (m/s)
1.4	4.35	1.63
1.2	4.73	1.61
1.0	5.02	1.68

### Material Properties

The aorta can be simply assumed to be a linear elastic material model in the range of material deformation, with a density of 1060 *kg*/m^3^, Young’s modulus of 4 MPa and a Poisson’s ratio of 0.45. TAV and BAV have been demonstrated to have similar fiber arrangement (Aggarwal et al., 2014) and can be modeled with the same material formulation. An incompressible Mooney–Rivlin hyperelastic model (Cao and Sucosky, 2017) was used, whose strain energy function can be defined as followed:

(1) $$\text{W}={\text{C}}_{10}\,\left({\bar{\text{I}}}_{1}-3\right)+{\text{C}}_{01}\,\left({\bar{\text{I}}}_{2}-3\right)+{\text{C}}_{11}\,\left({\bar{\text{I}}}_{1}-3\right)\,\left({\bar{\text{I}}}_{2}-3\right)$$

where ${\bar{I}}_{1}$ and ${\bar{I}}_{2}$ are the first and second deviatoric strain invariants and *C*_*10*_, *C*_*01*_, *C*_*11*_ are the material parameters from calibrated from *in vitro* tensile test data on porcine leaflet (Missirlis and Chong, 1978). All the parameters were shown in Table 2. In large scale blood flow, the blood can be modeled as weakly compressible Newtonian fluid, with density of 1050 *kg*/m^3^ and dynamic viscosity of 0.0035 Pa s. In explicit solution, this fluid material model uses equation of state to describe the pressure–volume relationship to represent the weak compressibility. Gruneisen equation of state was used in this study, which is defined as:

(2) $$\text{p}=\frac{\rho_{0}\,{\text{C}}^{2}\,\mu \,\left[1+\left(1-\frac{\gamma_{0}}{2}\right)\,\mu -\frac{a}{2}\,\mu^{2}\right]}{{\left[1-\left({\text{S}}_{1}-1\right)\,\mu -{\text{S}}_{2}\,\frac{\mu^{2}}{\mu +1}-{\text{S}}_{3}\,\frac{\mu^{3}}{{\left(\mu +1\right)}^{2}}\right]}^{2}}+\left(\gamma_{0}+\text{a}\,\mu \right)\,\text{E}$$

**TABLE 2:** Parameters of Mooney–Rivlin model for porcine leaflet material.

	C_10_	C_01_	C_11_
Values (kPa)	32.823	2.955	585.790

where ρ_0_ is the initial density of blood; C is the local sound speed of the blood and related to the compressibility of fluid. A value of 150 m/s was selected for C to reduce the computational cost according to previous researches (Mao et al., 2016); *S*_1_,*S*_2_,*S*_3_,γ_0_ are the constants in the equation, set as 1.9, 2.0, 0, 1.0, respectively; a is the first order volume correction coefficient, set as 5.0; E is the initial internal energy, set as 0 J; μ is the relative volume written as:

(3) $$\mu =\frac{\rho }{\rho_{0}}-1$$

where ρ is the current density of blood.

### Boundary Conditions and FSI Settings

In this work, the FSI between the blood and the aortic valve/aorta was calculated using the immersed boundary method (IBM). The IBM method was first proposed by Peskin (1972) to deal with the problem of heart valves. IBM method describes the fluid motion in a Eulerian view, and the fluid domain could be treated with static grid during the calculation process. The solid domain is immersed in the fluid domain. The force, velocity, and displacement at the interface could be exchanged by interpolation of adjacent nodes, which means that the fluid nodes do not need to coincide with the solid grid at the interface. IBM method had been demonstrated to be effective for FSI problems with large deformation (Marom, 2015; Bavo et al., 2016; Oliveira et al., 2019).

**FIGURE 4:**
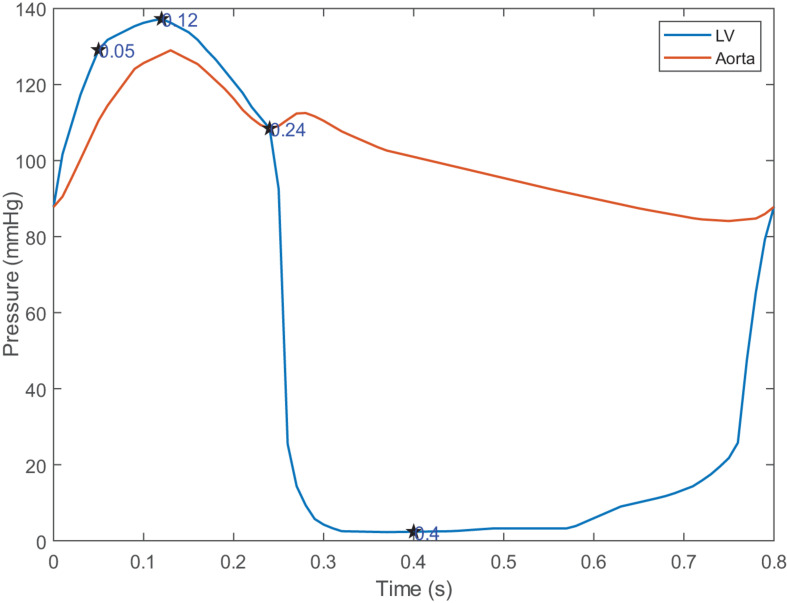
Pressure waveforms for boundary conditions and four characteristic moments.

For the solid domain, the aorta is subject to complex physiological constraints in human body, so both ends of the aorta was assumed to be completely fixed in this work. The attachment edges of the leaflets are connected with the tissue of the aortic sinus, and the leaflets will move following the aortic expansion. So, a tie constraint was employed between the attachment edges and the aortic root. For the fluid domain, two external solid components were introduced in inlet and outlet to apply the pressure boundary conditions which is shown in Figure 3C. Time-varying left ventricular and aortic pressure profiles were obtained from the literature (Mao et al., 2016). The pressure waveforms were shown in Figure 4. Both the aortic wall and the aortic valve surface were treated as a no-slip wall condition. An automatic surface-to-surface contact algorithm (Hallquist, 2007) with damping control and soft constraint was adopted to deal with the complex contact among the leaflets during the cardiac cycle.

All the FSI processes were completed using explicit solver provided by LS-DYNA R11.1.0 (LSTC, Livermore, CA, United States and ANSYS Inc., Canonsburg, PA, United States). The effectiveness of this software in heart valves problems had been widely confirmed in previous studies (de Oliveira et al., 2020; Luraghi et al., 2020). Two cardiac cycles (The duration of single cycle was set to 0.8 s) were considered in the calculation to eliminate the effect of the initial instability. The result of the second cycle was selected for data analysis. All model calculations were performed on an AMD Ryzen workstation of 3.7 GHz with Ryzen Threadripper 3970X CPU (64 processors) and 64 GB RAM, and the calculation time for each model was about 40 h.

### Hemodynamic Evaluation

Four moments (*t* = 0.05, 0.12, 0.24, 0.4 s) were chosen to describe the motion of the leaflets during the cardiac cycle, which is shown in Figure 4. These four moments represented maximum positive pressure difference (*t* = 0.05 s), peak systole (*t* = 0.12 s), beginning of diastole (*t* = 0.24 s), fully closure of aortic valve (*t* = 0.4 s), respectively.

Geometric orifice area (GOA) and effective orifice area (EOA) are the critical reference index for evaluating the performance of the aortic valve. GOA was measured by projecting the valve on the virtual valvular ring plane, which represented the dynamic changes of physical orifice area of the valve. EOA is a widely used indicator of aortic stenosis severity (Nishimura et al., 2014). According to the ISO-5840 Standard of Cardiovascular implants: cardiac valve prostheses (International Standard, 2013), EOA can be calculated as followed:

(4) $${\text{q}}_{V_{R\,M\,S}}=\sqrt{\frac{\int_{t_{1}}^{t_{2}}{\text{q}}_{V}\,{\left(t\right)}^{2}\,dt}{{\text{t}}_{2}-{\text{t}}_{1}}}$$

*q*_*V_RMS*_ is root mean square forward flow during the positive differential pressure period (ml/s). *q*_*V*_(*t*) is instantaneous flow at time (t). *t*_*1*_ and *t*_*2*_ is the time at start and end of positive differential pressure period, respectively. With these parameters, EOA is calculated as:

(5) $$\text{E}\,O\,A=\frac{{\text{q}}_{V_{R\,M\,S}}}{51.6\,\sqrt{\frac{△\,P}{\rho }}}$$

where ΔP is the mean pressure difference during positive differential pressure period (mmHg), ρ is the density of the fluid (g/cm^3^).

Besides, mean pressure difference and peak velocity are also important to represent the hemodynamic characteristics of BAV. Mean pressure difference were calculated between the cross-section of the virtual valvular ring and the beginning plane of ascending aorta during positive differential pressure period. Spatial-average pressure was used in cross-section. Peak velocity could be simply obtained from the post-processing. CO could be used to measure the aortic valve function under a specific cardiac work. Stroke volume was recorded in each model by calculating the accumulative fluid volume passing a reference plane (plane of the virtual annulus) in one cardiac cycle, and CO was the product of stroke volume and heart rate (75 bpm).

Strain on the aortic leaflet is considered to be an important factor of calcification progress (Halevi et al., 2018), so it can provide a valuable prediction for the location of aortic valve calcification. The aortic leaflets suffer higher pressure difference during diastole, and therefore it usually produces higher strain on the leaflet in diastole than in systole. Maximum principal strain on the leaflets was assessed in diastole.

## Results

### Morphology of Leaflets

**FIGURE 5:**
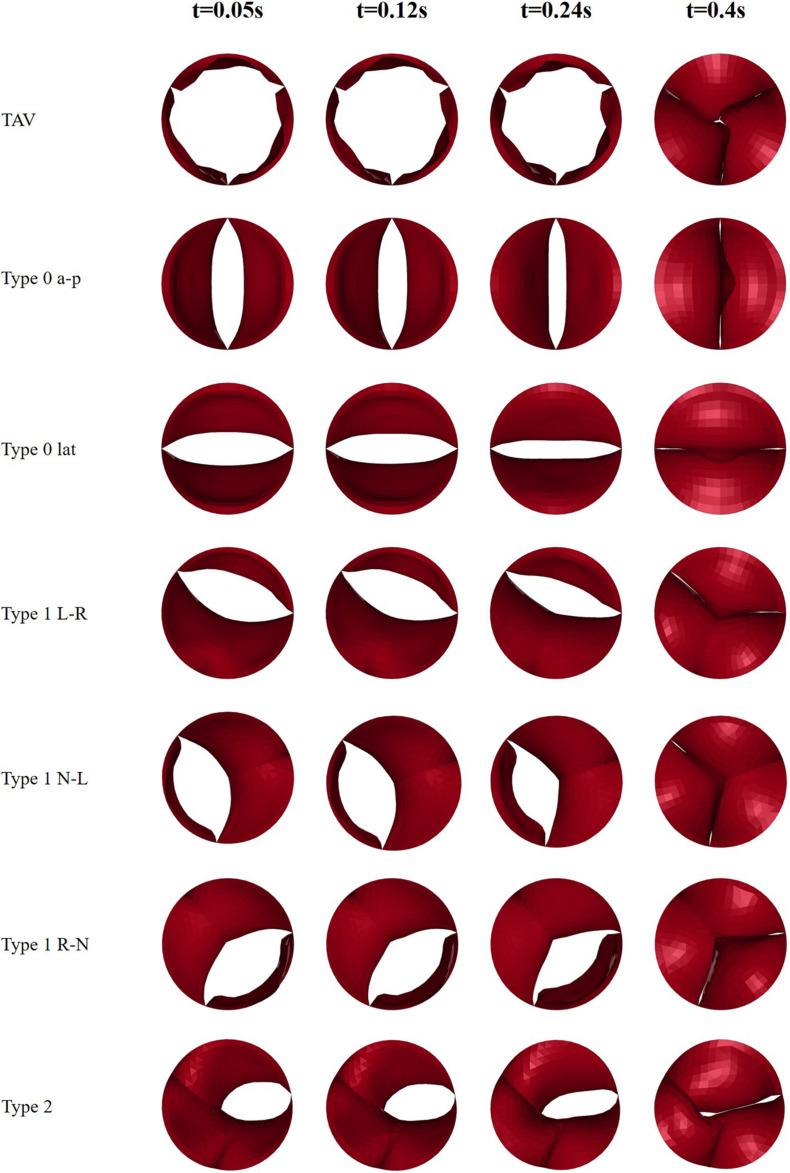
Snapshots of leaflets motion of TAV and BAV models at four moments (*t* = 0.05, 0.12, 0.24, 0.4 s).

This study recorded the dynamic change process of the leaflet morphology of TAV and BAV models during the cardiac cycle, and the results were shown in Figure 5. The aortic valve was fully opened at peak pressure difference moment (*t* = 0.05 s) and fully closed at diastole (*t* = 0.4 s). The orifice geometry of the normal TAV model was approximately circular, and that of Type 0 was long oval whose direction was consistent with the orientation of the valve. In Type 1 model, the opening amplitude of the non-fusion site was larger, and the fusion site was less affected by the raphe. In Type 2 model, the orifice was presented as a short oval.

**FIGURE 6:**
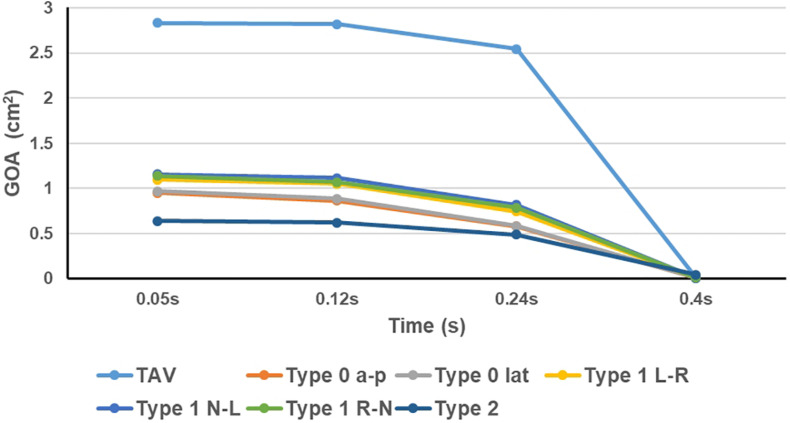
Dynamic variations of GOA of TAV and BAV models.

The GOA of the valve during the cardiac cycle represents the dynamic changes of the orifice of valve, and the results were shown in Figure 6. In all models, the GOA reached a maximum value at peak pressure difference moment (*t* = 0.05 s) and then gradually decreased. The peak value of GOA of each BAV phenotype was much lower than that of TAV, and Type 2 model showed the lowest GOA, which was only 23% of the TAV value. GOA of all BAV models at diastole (*t* = 0.4 s) was close to 0, and only Type 2 contributed to a maximum value of 0.044 cm^2^, which was five times that of TAV.

### Hemodynamic Performance

The calculation results of hemodynamic parameters of the TAV and BAV models were listed in Table 3. The CO of TAV was 4.73 L/min, which was near to 5 L/min of normal human physiological condition. Among all BAV models, Type 2 exhibited the minimum CO of 2.55 L/min, which was only 54% of TAV, while Type 1 R-N produced the maximum CO with 4.01 L/min, 85% of TAV. The average CO of Type 0 and Type 1 were 67 and 82% of TAV, respectively. No significant difference was shown between the subtypes of Type 0. However, the CO of Type 1 L–R was slightly lower than the other two Type 1 subtypes (about 7%).

**TABLE 3:** Hemodynamic results of TAV and BAV models.

	EOA (cm^2^)	CO (L/min)	ΔP (mmHg)	Peak velocity (m/s)
TAV	2.66	4.73	4.1	1.61
Type 0 a–p	0.79	3.15	20.4	2.98
Type 0 lat	0.84	3.18	20.4	3.03
Type 1 L–R	1.02	3.68	17.4	2.88
Type 1 N-L	1.06	3.98	17.1	2.84
Type 1 R-N	1.06	4.01	17.1	2.83
Type 2	0.56	2.55	24.5	3.35

Type 2 yielded the maximum value of mean pressure difference, ΔP (24.5 mmHg), which was about six times that of TAV. The ΔP of Type 0 a–p and Type 0 lat were both 20.4 mmHg, which was about five times of TAV. Type 1 N-L and Type 1 R-N generated the lowest ΔP in all BAV models, about 424% increase compared with TAV. Subtypes of Type 1 BAV predicted similar ΔP, regardless of raphe position.

Effective orifice area characterizes the smallest cross-sectional area of the jet flow downstream of the aortic valve (Garcia and Kadem, 2006). Among all BAV models, both Type 1 N-L and Type 1 generated the maximum EOA with a value of 1.06 cm^2^, while Type 2 exhibited the minimum of 0.56 cm^2^. In general, EOA of Type 0 subtypes and Type 1 subtypes was similar and no significant difference was found.

Compared with TAV, BAV generated a higher peak velocity. Type 2 was associated with a maximum peak velocity of 3.35 m/s, which was 108% higher than that of TAV. The difference in peak velocity between Type 0 and Type 1 subtypes was not significant, and the average peak velocity of Type 0 and Type 1 subtypes increased by 87 and 77%, respectively compared with TAV.

### Flow Patterns

**FIGURE 7:**
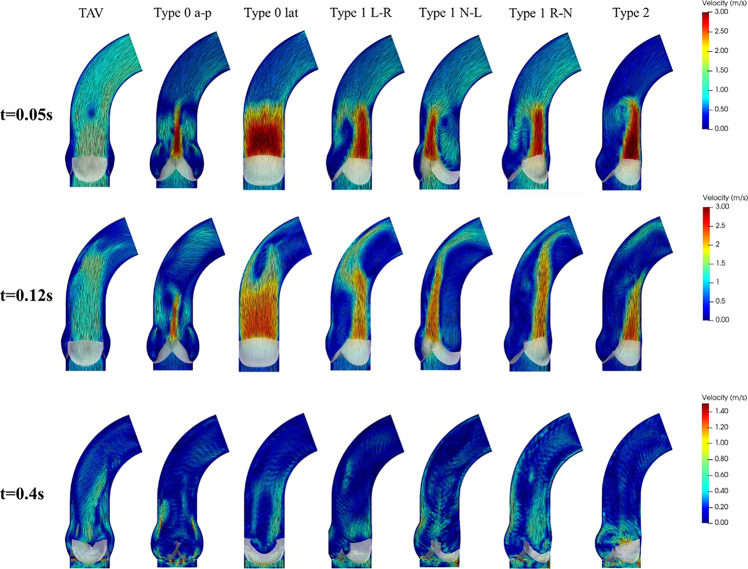
Flow patterns and streamlines of TAV and BAV at some characteristic moments (*t* = 0.05, 0.12, 0.4 s).

Compared with the symmetric flow profile of TAV, BAV models generated an asymmetric flow pattern with strong jet flow effect during systole, as shown in Figure 7. Type 0 model was related to a narrow jet flow profile with central symmetry, and the jet flow orientation was consistent with Type 0 subtypes. Type 1 BAV exhibited a clear eccentric jet flow, and the jet flow direction was associated with position of the raphe. In Type 1 L–R, Type 1 R-N, and Type 2 models, the blood flow mainly impacted the left side of the ascending aorta, while the impact location was the right side in Type 1 N-L model. Unidirectional flow was observed in ascending aorta in TAV model, while BAV models would form strong vortices at the end of the jet area. Type 2 showed the most obvious vortex effect, and the forward flow along the aorta was severely blocked. During diastole, the flow fields of TAV and BAV models were relatively disturbed. Small vortices appeared near the closed leaflets. Local region with higher flow velocity was found in Type 2 model, which indicated that a slight regurgitation phenomenon occurred, and a large vortex region was also generated in the downstream direction of the valve.

### Maximum Principal Strain of Leaflets

**FIGURE 8:**
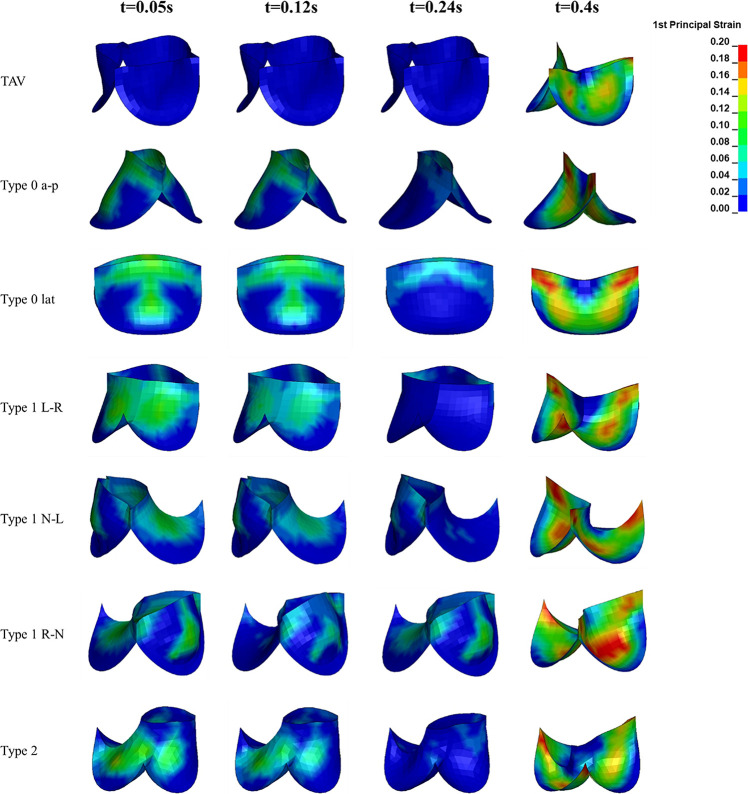
The leaflet strain distribution of TAV and BAV during cardiac cycle (*t* = 0.05, 0.12, 0.24, 0.4 s).

**FIGURE 9:**
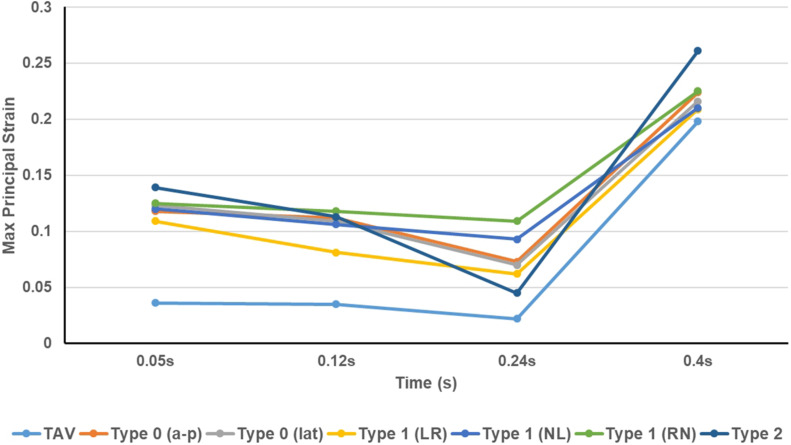
The peak value of maximum principal strain of TAV and BAV models at different moments (*t* = 0.05, 0.12, 0.24, 0.4 s).

The maximum principal strain distribution and peak strain of the leaflets at different moments were shown in Figures 8, 9, respectively. At maximum positive pressure difference moment during systole (*t* = 0.05 s), the peak value of the Maximum principal strain of BAV was much higher than that of TAV, and the peak strain of Type 2 was the highest, which was 3.9 times that of TAV. The high strain area of BAV was mainly distributed in the center of the leaflet and near the free edge. After the maximum positive pressure difference moment, the strain of TAV and BAV decreased gradually, and then increased rapidly in the diastolic phase. When the valve was completely closed (*t* = 0.4 s), the strain on leaflets was much higher than that in systole. The peak strain of the leaflets of BAV during diastole was higher than that of TAV. The diastolic strain difference for Type 0 and Type 1 was less than 10%, and the difference was also small compared with TAV model. Type 1 L–R was associated with the lowest peak strain in BAV models, which was 6% higher than TAV, while Type 2 BAV exhibited the highest peak strain, 30% higher than TAV. Besides the increase of peak strain, the region of high strain (>0.15) in BAV model in diastole was more extensive than that in TAV model. The high strain regions of TAV and Type 0 models were mainly concentrated at the junction of commissure edge and free edge, but the distribution area of Type 0 BAV was larger and extended to the central region of leaflets. The high strain regions of Type 1 and Type 2 occurred at the commissure edge and the junction of raphe and aortic root, as well as the middle region of leaflets.

## Discussion

In this study, three-dimensional idealized models of all BAV phenotypes were constructed and FSI method (IBM) was applied to quantitatively study the hemodynamic performance of TAV and all BAV phenotypes, including CO, EOA, peak velocity, and mean pressure difference. In addition, GOA and Maximum principal strain on leaflets were measured to describe the dynamic orifice variations of leaflets and evaluate the durability of the aortic valve, respectively. The results of this study indicated phenotype-associated congenital stenosis and abnormal flow for all BAV models, accompanied by higher mean pressure difference, peak velocity, and leaflet strain.

### Differences in Hemodynamic Characteristics

This study presented abnormal hemodynamic performance for all BAV phenotypes. In the positive pressure difference phase, the mean pressure difference of BAV was four times higher than that of TAV, and Type 2 BAV was associated with the largest pressure difference, which was as high as six times that of TAV. From Bernoulli equation, the higher mean pressure difference indicated a rapid GOA decrease across the valve, which usually corresponded to the occurrence of stenosis. The CO and EOA of BAV were lower than those of normal TAV. Type 2 BAV was the phenotype with the worst hemodynamic performance. According to the criteria of clinical diagnosis (Grimard and Larson, 2008; Nishimura et al., 2014), all BAV phenotypes could be diagnosed as aortic stenosis, among which Type 1 was moderate aortic stenosis, and Type 0 and Type 2 were severe aortic stenosis. Our result demonstrated a negative impact on CO and EOA of BAV, which prevented the blood supply from the left ventricle to the aorta. These findings were consistent with other computational results (Cao and Sucosky, 2017) and clinical statistics (Akins et al., 2008; Grimard and Larson, 2008). But in fact, not all BAV patients will exhibit symptoms related to insufficient blood supply, which can be explained by the Starling compensation mechanism, where the heart will increase its own work to improve CO to satisfy the blood supply needs of the body. Therefore, from the view of the long-term development of the disease, BAV will increase the burden of the heart, which may cause serious heart diseases such as myocardial thickening, cardiac hypertrophy, and heart failure.

In addition to the perfusion of aorta, BAV also affected the systolic jet flow and the flow patterns in aorta. BAV resulted in the asymmetry of the flow pattern and the great rise of peak velocity, and the jet flow direction was consistent with the orientation of BAV.

Obvious eccentric jet flow was found in Type 1 and Type 2 BAV models, which made a strong impact on the ascending aortic wall. The long-term effect of the high-speed eccentric jet on the ascending aorta may be an important reason for its expansion (Cao et al., 2017; Liu et al., 2018; de Oliveira et al., 2020). Although the flow profile of Type 0 was roughly centrosymmetric, the long narrow jet at both ends of the cross-section would also generate higher local stress on the ascending aorta. In fact, Type 0 was also clinically accompanied by the appearance of ascending aortic dilation (Cao et al., 2017). Besides, BAV produced a strong vortex in the aorta at the systole, blocking the blood flow downstream. During diastole, the results showed a large vortex above the valve and slight regurgitation of Type 2 BAV. It meant that BAV malformation not only made an important impact on hemodynamics during systole, but also altered the hemodynamic performance during diastole.

### Differences in Valve Mechanical Characteristics

Affected by the structure of the aortic valve, the opening of BAV during systole was impeded. It could be observed from the dynamic changes of GOA that BAV would close the valve more quickly than TAV. At beginning of diastole when the pressures were equal on both sides of the valve (*t* = 0.24 s), the GOA of BAV decreased more greatly (TAV decreased by 10%, Type 0 decreased by about 40%, Type 1 decreased by about 30% on average, and Type 2 decreased by 23%). Smaller GOA and quicker valve closure are the direct causes of the decrease in BAV CO.

The strain on the aortic valve is an important index to evaluate the mechanical performance of the valve. The results of this study showed that the Maximum principal strain presented a trend of weakly decreasing first and then increasing sharply in the cardiac cycle, and bearing highest strain during diastole, regardless of TAV or BAV. Owing to the fact that its structure hindered the opening of the valve and caused the high pressure of the ventricle to directly act on the surface of the leaflets, the strain of BAV during systole was much higher than that of TAV. The peak strain of BAV during diastole also increased, accompanied with a wider high-strain region. This phenomenon may be related to the shape of the BAV leaflets which is not conducive to share the pressure difference uniformly. The abnormal structure of BAV causes its strain to increase throughout the cardiac cycle, and the long-term high strain is considered to be one of the inducing factors of aortic calcification (Hutcheson et al., 2012). Therefore, the malformation of BAV may accelerate the calcification process of aortic valve (Cao and Sucosky, 2017), thereby further aggravating aortic stenosis.

### Assumptions Rationality and Limitations

In this study, several assumptions on model settings and materials were made to reduce the complexity. Some limitations were as followed. Firstly, parameterized idealized models based on physiological anatomy were used instead of the patient-specific model. Although patient-specific models could actually reflect the morphology and structure of diseased valves and aortic roots, the morphological differences between individuals were huge and the sizes varied (Cao and Sucosky, 2015), making it difficult to quantitatively compare the differences between BAV phenotypes. So, in order to highlight the hemodynamic differences between various BAV phenotypes and reduce the impact of other factors in models, a parameterized model was adopted. Key parameters such as annulus diameter, valve height, commissure edge height, and aortic length were kept consistent.

Geometric orifice area characterizes the dynamic changes of the physical orifice area of the aortic valve, and the maximum value appeared at the moment when the pressure difference was greatest (*t* = 0.05 s). In this study, the maximum GOA of the TAV and BAV models during systole was lower than the GOA reported in similar studies (Cao and Sucosky, 2017; de Oliveira et al., 2020), which might be due to the differences in the models. Diameter of the aortic annulus used in this article was 23 mm, which was a smaller annulus size. Simultaneously, raphes consistent with physiological anatomy were added to the model, which hindered the opening of the BAV.

In the calculation process of FSI, we used physiological left ventricular pressure and aortic pressure as the boundary conditions of the fluid domain and apply them to all BAV models. In fact, the left ventricular pressure of BAV patients may be higher than healthy people with normal TAV, which is different from our assumption. In other similar studies, common flow condition was used in the left ventricular inlet (de Oliveira et al., 2020), ignoring the influence of BAV malformation on CO, which also deviated from the actual physiological situation. In our study, we used the pressure value and waveform corresponding to the healthy TAV as the inlet pressure of the BAV model, with the purpose of comparing the CO provided by BAV models under the same left ventricular work. The results showed a much lower CO of BAV compared with the normal TAV model under the same pressure conditions, which also indicated that the heart may improve the blood supply capacity of the heart through structural or functional adjustments after BAV disease.

In this study, the blood flow was assumed to be laminar, and the effect of turbulence was not considered in the calculation. According to the other scholars’ study, the Reynolds number of the flow in aorta could reach 3000–3900 during cardiac cycle (Formaggia et al., 2010), which is in the transitional stage of turbulence transition. For the BAV models, abnormal valve structure may lead to the formation of turbulence. At present, it is very difficult for the existing turbulence model to be applied to the FSI problem, especially large deformation is involved during the dynamic changes of the leaflets. At the same time, the introduction of the turbulence model would greatly increase the computational cost of FSI calculation. In addition, the WSS and OSI of the ascending aorta may be the important indicators to evaluate the risk of ascending aortic dilation (Cao and Sucosky, 2017), but the calculation and analysis of these two parameters were lacking in current study due to the dual limitations of the IBM method and the grid size of the fluid domain, which resulted in insufficient physical information captured on the aortic wall. Accurate capture of WSS and OSI requires a higher grid density, which will also bring a huge increase in computational cost. Therefore, this study only used the direction of the jet flow and the flow distribution to predict the potential position of the ascending aortic dilation indirectly. The evaluation method needs to be improved in future work.

In the simulation study, the material of aortic valve was assumed as an isotropic material to avoid the problem of excessive distortion of the mesh during the contact process of the leaflets. In fact, the aortic valve tissue exhibits obvious fiber arrangement (Grande-Allen et al., 2001; Feng et al., 2020), which belongs to an anisotropic material. Studies have shown that the isotropic and anisotropic leaflet materials may have little effect on the study of hemodynamic performance (Weston et al., 1999; Ge and Sotiropoulos, 2010). However, for the study of the mechanical properties of the valve, including the stress and strain on leaflets, it may be necessary to consider the anisotropic constitutive model. In addition, the same material properties were adopted for TAV and BAV models, which is a reasonable assumption based on the similarity of the fiber arrangement of leaflets for them. However, it is still unclear whether the material properties will change because of the BAV malformation.

### Clinical Significance and Application Value

This study demonstrates the importance of BAV classification for the clinical prediction, diagnosis, and treatment of BAV-associated diseases because of the different hemodynamic characteristics (including the mean pressure difference, peak flow velocity, CO, EOA, and flow field) and valve mechanical properties (including GOA dynamic changes and Maximum principal strain of leaflets) of BAV phenotypes. CO and EOA are often used to clinically evaluate the degree of aortic stenosis to determine whether surgical intervention is required (Baumgartner et al., 2009). The Maximum principal strain on the leaflet can be used to predict the progress and potential location of the calcification. The results of our research show that the performance of the valve in diastole may have a more important impact on its long-term performance than systole. This is also an important clinical concern, which was ignored in previous studies (de Oliveira et al., 2020). In particular, our results suggest that Type 2 may be associated with slight regurgitation during diastole compared with other BAV phenotypes. Moreover, the various evaluation indexes of Type 2 are the worst among all phenotypes. Therefore, it may need to pay more attention to Type 2 BAV in actual clinical practice.

## Conclusion

In this study, a workflow of FSI modeling and simulation for aortic valves are established. Based on idealized models, the hemodynamic characteristics and mechanical properties of BAV with different phenotypes throughout the cardiac cycle are systematically analyzed. Among all BAV phenotypes, the hemodynamic performance of Type 1 is the best. The stenosis degree of Type 1 L–R is slightly higher than that of the other two subtypes. The potential aortic dilation position of different subtypes is related to the jet flow direction, which is consistent with the leaflet direction. The stenosis degree of type 0 is higher than that of Type 1. Type 0 models shows symmetrical flow patterns, and no obvious hemodynamic difference is observed between its subtypes except jet flow direction. Type 2 exhibits the most severe stenosis, whose potential location of dilation may be above the left-coronary sinus and non-coronary sinus. Slight regurgitation also occurs during diastole in Type 2 phenotype. During diastole, the leaflets of BAV models suffered higher strain compared with TAV. The peak strain of Type 2 is the highest, which may accelerate the development of calcification and other pathologies. No significant strain difference is presented between Type 0 and Type 1. These findings reveal the specificity of the pathological phenotypes of BAV, which has important guidance for the clinical diagnosis and treatment of BAV.

## Data Availability Statement

The original contributions presented in the study are included in the article/supplementary material, further inquiries can be directed to the corresponding author/s.

## Author Contributions

WY designed the research proposal, conducted the data analysis, and wrote the manuscript. JL established the model and did the numerical simulation. WW provided the clinical data and assisted in data analysis. LW proposed clinical research directions and modified the manuscript. SW designed the research proposal, contributed to manuscript modification, and supervised the overall project. All authors read and edited the final version of the manuscript.

## Conflict of Interest

The authors declare that the research was conducted in the absence of any commercial or financial relationships that could be construed as a potential conflict of interest.

## Publisher’s Note

All claims expressed in this article are solely those of the authors and do not necessarily represent those of their affiliated organizations, or those of the publisher, the editors and the reviewers. Any product that may be evaluated in this article, or claim that may be made by its manufacturer, is not guaranteed or endorsed by the publisher.
